# The impacts of the Covid-19 pandemic on surrogacy in India: The role of social work

**DOI:** 10.1177/1473325020981082

**Published:** 2021-03

**Authors:** Lopamudra Goswami, Stephen Anthony Larmar, Jennifer Boddy

**Affiliations:** School of Human Services and Social Work, Department of Health, Griffith University, Gold Coast, Australia; School of Human Services and Social Work, Department of Health, Griffith University, Meadowbrook, Australia; School of Human Services and Social Work, Griffith University, Parklands Drive, Australia

**Keywords:** Disasters, marginality, mothering, surrogacy, Covid-19, lockdown, Pandemic

## Abstract

The impacts of the Covid-19 pandemic have been catastrophic internationally, with alarming rates of cases and deaths, as well as travel bans and countrywide lockdowns. While many industries are experiencing the deleterious effects of Covid-19, international surrogacy is facing enormous ethical challenges resulting from the pandemic. Drawing on the first author’s reflections on research with Indian surrogate mothers, coupled with contemporary literature, this paper highlights the impacts of Covid-19 on surrogacy in India, particularly regarding the strict lockdown laws intended to protect civil society. This paper discusses the serious issues facing key actors involved in surrogacy, including surrogate mothers and commissioning parents. Focus is given to the psychological impacts on newborn babies caught in a liminal space as a result of lockdown laws. The authors conclude with reflections on the role of social work in protecting women and children in international surrogacy, particularly during a pandemic.

## India in lockdown: The current context

The first lockdown in India commenced on March 25 and spanned a 21-day period. However, it became apparent that it would need extending as daily cases increased nationally. On April 14, the lockdown extended for another 19 days and a further 14 days on May 1 (Ojha, 2020). Based on media reports and anecdotal data, the lockdowns have deeply impacted the Indian economy leading to millions of job losses (Choudhury, 2020). The most impacted workers are women who comprise less than a quarter of the labour force, despite being 49% of the country’s population, earning an average of 35% less compared to men (Beniwal, 2020).

## Surrogate mothers in India: Before and during Covid-19

Surrogacy in India is a survival strategy for many women living in poverty (Pande, 2009a). Surrogates engage in the process to provide a better future for themselves and their children (Kroløkke and Pant, 2012). However, many surrogate mothers experience physical and emotional pain, economic hardship and a sense of loss (Rotabi et al., 2017).

Owing to factors commonly associated with destitution, surrogate mothers in India are seriously exploited and ill-informed about the process and legal arrangements associated with surrogacy (Deomampo, 2016). As highlighted in the first author’s prior research, such exploitation emphasises the power imbalance between surrogate mothers and agencies facilitating the surrogacy process (Rotabi et al., 2017). Surrogate mothers must meet specific requirements outlined by the Indian Council of Medical Research (ICMR) (clause 3.10.5) to be declared fit for the procedure (Kotiswaran, 2019). Ironically, despite such regulation, few laws exist advocating for the rights of surrogate mothers in India (Kotiswaran, 2019). While Indian surrogate mothers are conceptualised as the ‘perfect mother-worker’ (Pande, 2010a), no consideration is given to the impacts of potential attachment to the child for the surrogate mother during or after the period of gestation or to her wellbeing as she engages in the process. Within the context of the first author’s current research, the attachment needs of surrogate children and the wellbeing of mothers is given little consideration necessitating the role of support workers such as social workers in advocating for the needs of this vulnerable group.

Surrogate mothers in India usually live in surrogacy clinic assigned hostels during their pregnancies (Rudrappa, 2015). This arrangement facilitates supervision of the surrogate’s health and medical needs undertaken by medical staff and commissioning parents to ensure the safety of the unborn child (Pande, 2009a). While this practice raises questions concerning infringement of a surrogate mother’s rights under normal circumstances (Carney, 2011), with the Indian lockdown, there are serious concerns about surrogate mothers’ access to basic services including health care, food and transportation. Both research and practice focusing on social work’s role in facilitating community support for this vulnerable population is even more critical during this pandemic period.

The first author has observed that the lockdown has presented many challenges impacting on the care of surrogate mothers. For example, clinics are not able to prioritise the physical or psychological safety of surrogate mothers who already have compromised health and wellbeing. From the first author’s prior and current fieldwork reflection, surrogate mothers travel to hospitals and clinics regularly for medical support throughout the period of gestation (Pande, 2010a; Rotabi et al., 2017). Agents who mediate between surrogate mothers and health clinics often oversee such interventions. A serious ethical issue is that many of these agents comprise illiterate or non-English speaking women, thereby igniting concerns over their oversight of medical procedures without appropriate training and knowledge. It is clear that greater advocacy for the coordination of services for surrogate mothers is critical at this point in time.

Given the range of medical procedures administered for surrogate mothers during the surrogacy period, measures need to be taken to ensure that surrogate mothers avoid contracting Covid-19. This is of particular concern given reliance on public transport and communal accommodation requiring surrogate mothers to engage in close proximity with others without safely controlled social distancing. The authors contend that community support for surrogate mothers must include clear delineation of medical and social distancing measures to protect this population.

## The plight of commissioning parents

Since the lockdown period, doctors employed in surrogacy clinics formally documenting birth rates indicated that a small percentage of delivered babies were handed over to commissioning parents (Raja, 2020). Of these parents, it has been reported that they could access flights prior to lockdown to travel to the surrogacy destination (Banerjee and Pandey, 2020). Other commissioning parents have risked their health and safety, travelling vast expanses via motor vehicle to be united with their surrogate babies (Ojha, 2020; Raja, 2020). In one case, commissioning parents were able to collect their surrogate daughter through air ambulance from Surat to Bangalore (*NDTV*, 2020). However, the number of babies unable to be united with their respective commissioning parents is concerning. Available reports indicate that babies either remain in hospitals or are taken into care by assigned relatives (Raja, 2020). Of the remaining babies, there are serious concerns about the limited capacity of medical personnel to address each child’s physical and psychological health. The first author of this paper has observed through her current research into Indian surrogacy that, while some commissioning parents were in a position to collect their surrogate children, others with fewer resources were circumstantially prevented from being united with their children. It is our view that it is of paramount importance that the Indian government provides assistance in this time of crisis.

## A further silencing of the Indian surrogate mother’s voice

Given the situations facing stranded Indian surrogate mothers and babies in the midst of the Covid-19 pandemic, insight is critical to understand how to provide timely support. At the point this article was written, one report had been published in India (Raja, 2020) focusing on an Indian couple living in the U.S. prevented from meeting their surrogate baby due to the international travel ban. The report gave no information about the surrogate mother, discharged after 15 days in hospital, with the care of the baby entrusted to the hospital’s nurses. No insight was given to the surrogate mother’s involvement postnatally. If the surrogate mother provided antenatal care, questions arise regarding the woman’s right to reasonable compensation. In contexts such as the Ukraine, commissioning parents pay for postnatal care including payment for nursing support (Nobre, 2020). This raises questions about what is happening in India, including the rights of surrogate mothers. Our reflections of the plight of these children highlight the need for transparent dissemination of information and for trained social workers to fill this current gap in supporting surrogate mothers and children during the Covid-19 pandemic.

## Who is parenting the surrogate baby in India during Covid-19?

There are no current available statistics documenting the number of stranded surrogate babies in the care of nurses or relatives. The most recent estimate of surrogate babies being born per year in India sits within the range of 300 to 3000 (Deomampo, 2016). However, the likely number of babies born yearly is in excess of 6000 (Kotiswaran, 2019). While reports from the U.S. and Ukraine indicate that in some cases, surrogate mothers are looking after their surrogate children (Grytsenko, 2020), no data is available in India to support a similar trend. Based on current circumstances, it is plausible that this population of children may be highly neglected or unattended; not necessarily as a result of any unsympathetic attitudes of caregivers, but rather, due to the lack of suitable training, personnel or resources available. The authors argue that supportive interventions involving the work of social workers, such as development of temporary clinics and/or coordination between existing services to strengthen current responses in addressing the needs of surrogate children is warranted during this time.

## The needs of the surrogate child

Research is clear that infant-mother attachment is critical to the healthy development of the child (McElwain and Booth-LaForce, 2006). Healthy attachment is not developed instantaneously; rather, it involves continued interaction between mother and child post-delivery (Bayne and Kolers, 2003). As a result of Covid-19 in India, newborn surrogate children are commencing their lives without the care necessary for secure attachment. This is made increasingly complex by the sudden removal of the child from the surrogate mother, creating a rupture in the child’s attachment (Bandelli, 2019). We are of the view that these factors raise serious concerns about future attachment between the surrogate baby and commissioning parents. From an adoption-based discourse, abandoned children moving between families experience difficulties in forming healthy attachment in relationships (Serbinski, 2017), building identity, and dealing with fear of rejection in later life (Lemieux, 2016). This discourse provides an appropriate lens to understand the influence of contextual factors associated with the Indian lockdown on the physical and psychological health of surrogate children raised by a spectrum of individuals for an extended period of time. As mentioned earlier, the role of social workers in supporting these vulnerable children is critical. Social workers are well placed to engage with both government and non-government services to develop innovative health care initiatives to close the gaps in service provision for surrogate children during the Covid-19 lockdown.

## Implications for social work practice – Authors’ reflections

Social work is grounded in a commitment to social justice and human rights, with a primary aim of overcoming oppression and marginalisation. Consequently, we as social workers have a key role to play in addressing the inequalities and injustices experienced by Indian surrogate mothers and children, especially during the Covid-19 pandemic. In particular, social workers such as the authors’ researching in the fields of assisted reproduction, maternal health, and women and child’s rights should be advocating for the rights of surrogate mothers and children by lobbying the government and drawing attention to key issues. They should also be working with organisations to ensure surrogate mothers’ and children’s medical and emotional needs are met post-delivery and that measures are taken to prevent a surrogate mother or child from contracting Covid-19. This will involve, in part, working with organisations to draft and administer social distancing and hygiene policies in clinics and hostels. Further, at present, surrogate mothers have very few opportunities to express their concerns. Social workers in India should partner with organisations to provide avenues for surrogate mothers to give feedback and have it addressed. This is particularly important during this unprecedented time. It is apparent that greater attention is needed to address the needs of surrogate mothers and children during the Covid-19 pandemic. Social workers in India, and across the globe, are well positioned to argue for the rights of surrogate mothers and children, and ensure their voices are heard.

## Conclusion

The current situation in India for surrogate mothers, babies and commissioning parents calls for urgent intervention within civil society to provide necessary measures to safeguard the needs of this already vulnerable population. The rights of surrogate mothers and babies must be at the forefront of laws and policies to ensure they are duly upheld. The role of qualitative research to understand the lived experiences of this population is critical. Further, the implementation of community based supportive interventions including the work of social workers for this population is becoming increasingly apparent with the emerging issues exacerbated during the pandemic lockdown. It is critical that such interventions are designed in consultation with women engaged in surrogacy and health professionals with experience in the sector. Finally, immediate action must be taken to address the attachment needs of surrogate children to ensure their ongoing physical and psychological health.
